# Attitudes, knowledge, and preferences of the Israeli public regarding the allocation of donor organs for transplantation

**DOI:** 10.1186/s13584-020-00376-3

**Published:** 2020-05-04

**Authors:** Amir Elalouf, Joseph S. Pliskin, Tehila Kogut

**Affiliations:** 1 Department of Management, Bar Ilan University, 5290002 Ramat Gan, Israel; 2 Department of Industrial Engineering and Management and Department of Health Systems Management, Ben Gurion University of the Negev, Beer-Sheva, Israel; 3 Department of Health Policy and Management, Harvard T.H. Chan School of Public Health, Boston, MA USA; 4 Department of Education & Decision Making and Economic Psychology Centre, Ben Gurion University of the Negev, Beer-Sheva, Israel

**Keywords:** Organ donations, Public preferences, Kidney transplantation, Allocation policy, Medical efficiency, Equity

## Abstract

**Background:**

There is a stark disparity between the number of patients awaiting deceased-donor organ transplants and the rate at which organs become available. Though organs for transplantation are assumed to be a community resource, and the organ supply depends on public willingness to donate, current allocation schemes do not explicitly incorporate public priorities and preferences. This paper seeks to provide insights regarding the Israeli public’s preferences regarding criteria for organ (specifically, kidney) allocation, and to determine whether these preferences are in line with current allocation policies.

**Methods:**

A market research company administered a telephone survey to 604 adult participants representing the Jewish-Israeli public (age range: 18–95; 50% male). The questionnaire comprised 39 questions addressing participants’ knowledge, attitudes, and preferences regarding organ donation and criteria for organ allocation, including willingness to donate.

**Results:**

The criteria that respondents marked as most important in prioritizing waitlist candidates were maximum medical benefit (51.3% of respondents) and waiting time (21%). Donor status (i.e., whether the candidate is registered as an organ donor) was ranked by 43% as the least significant criterion. Most participants expressed willingness to donate the organs of a deceased relative; notably, they indicated that they would be significantly more willing to donate if organ allocation policies took their preferences regarding allocation criteria into account. Unlike individuals in other countries (e.g., the UK, the US, and Australia) who responded to similar surveys, Israeli survey respondents did not assign high importance to the candidate’s age (24% ranked it as the least important factor). Interestingly, in some cases, participants’ declared preferences regarding the importance of various allocation criteria diverged from their actual choices in hypothetical organ allocation scenarios.

**Conclusions:**

The findings of this survey indicate that Israel’s citizens are willing to take part in decisions about organ allocation. Respondents did not seem to have a strict definition or concept of what they deem to be just; yet, in general, their preferences are compatible with current policy. Importantly, participants noted that they would be more willing to donate organs if their preferences were integrated into the allocation policy. Accordingly, we propose that allocation systems must strive to respect community values and perceptions while maintaining continued clinical effectiveness.

## Introduction

**Fig. 1:**
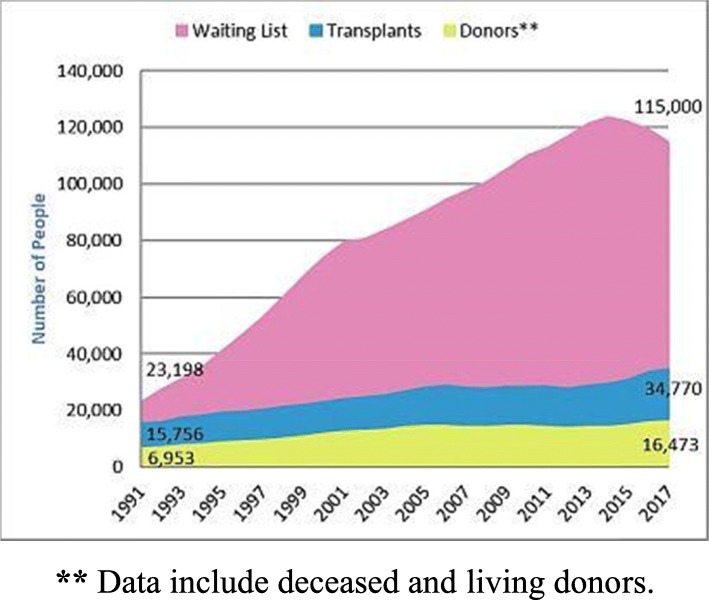
Organ shortage in the United States [1], ** Data include deceased and living donors

Organs for transplantation are a scarce resource. In many countries, there is a stark disparity between the number of patients awaiting deceased-donor organ transplants and the rate at which organs become available (for example, Fig. 1 portrays the organ shortage in the United States, whereas Table 1 depicts the number of waiting list candidates for each organ in Israel). A key factor in this disparity is scarcity of registered donors. For example, recent reports by the US Organ Procurement and Transplantation Network (OPTN) indicate that 95% of US adults support organ donation, yet only 54% are actually registered as donors [3]. In a recent survey administered in the UK, two out of three respondents expressed willingness to donate organs, yet only a third had signed up for the organ donation register [4]. In Australia, as of April 30, 2019, the total number of registered donors was 6,737,042 out of 24.5 million adults in the population (approximately 27%) [5]. Israel’s rates of donor card signees are among the lowest in the developed world. Only 14% (944,849) of adults have signed an organ donor card [6].

**Table 1:** Waiting list candidates in Israel [2]

Year*	Kidneys	Liver	Heart	Lungs	Heart and lungs	Kidney and pancreas	Total waiting list candidates	Increase/decrease in the % of waiting patients	Israeli population (Millions)	% of waiting patients in Israel
2010	690	151	133	66	6	23	1069		7.624	0.014%
2011	733	159	128	79	2	16	1117	+ 4.3%	7.766	0.014%
2012	729	135	96	70	1	10	1041	−7.3%	7.91	0.013%
2013	755	164	93	90	1	11	1114	+ 6.55%	8.06	0.014%
2014	762	124	89	87	2	11	1075	−3.6%	8.216	0.013%
2015	849	146	73	70	4	18	1160	+ 7.3%	8.38	0.014%
2016	843	131	73	83	6	17	1153	−0.6%	8.546	0.013%
2017	847	104	63	89	6	7	1116	−3.32%	8.712	0.013%
2018	840	110	74	102	6	6	1138	+ 1.93%	8.84	0.013%
2019	813	101	85	109	5	10	1123	−1.34%	9.1	0.012%

*As of January 1st of each year. Only active candidates are mentioned

**Fig. 2:**
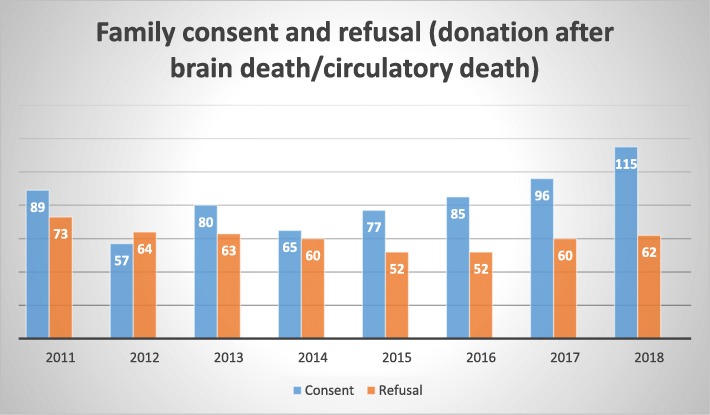
Familial consent in Israel [8]

An additional factor that has an immense effect on organ availability is family consent rate [7]. When a family does not provide consent for a deceased relative to serve as a donor—even if the deceased has declared a wish to do so—conventional medical practice is to refuse to retrieve that individual’s organs and tissues. Thus, families can prevent the use of viable organs. Although familial consent rate has noticeably increased over the years, the percentages are still far from satisfactory. Figure 2 illustrates the number of families in Israel (from 2011 until 2018) that gave their consent or refused to donate the organs of a deceased relative.

In light of the shortage of organs and the long waiting times for transplantation (the average waiting time for a kidney in Israel is 8 years, while in the US the average time frame is 5 years) [9, 10], policy-makers, in Israel and abroad, face not only the challenge of increasing the availability of donor organs but also the challenge of determining the criteria by which existing organs should be allocated. In defining allocation policies, decision-makers tend to incorporate a complex set of considerations, including medical need, medical urgency, capacity to benefit, donor/recipient matching, and logistical factors [11]. Though the details of organ allocation policies may differ across countries, these policies—each in its own way—generally strive to strike a balance between two encompassing principles: medical efficiency (or utility), i.e., the idea that each organ should be transplanted into the recipient in whom it will survive the longest [12], and the principle of equity, which demands that all persons who would benefit from a transplant should have comparable opportunities to receive one [13, 14]. It is important to realize that it is inherently impossible to simultaneously maximize medical efficiency and equity. Rather, there is a trade-off between the two principles, and this trade-off has been at the focus of ongoing philosophical and ethical debates for many years.

The current study focuses on an element that remains noticeably absent from this debate and from the allocation decisions made in practice: namely, public perceptions and opinions regarding organ allocation criteria. Owing to the fact that the organ supply depends on public willingness to donate, and given that the organ shortage can be defined as a public health problem or at least a critical challenge, it can be assumed that deceased-donor organs for transplantation are a community resource or public goods. In other words, people have a shared interest in ensuring that donor organs are available to all, and thus would be better off cooperating with regard to the provision and allocation of such organs. Therefore, it seems that allocation policies should, to some extent, consider community preferences regarding which factors should be prioritized in allocation decisions [15]. Though this notion has gained some traction in recent years [16], current allocation schemes do not explicitly incorporate community preferences. Indeed, it seems that very little precise information on such preferences is available at all.

It is worth mentioning that, in the US and in the UK, the public does have a formal role in health-related policy-making. In particular, in the US, the public can comment and provide feedback on policy proposals put forward by the US Health Resources and Services Administration. Moreover, the public’s comments and remarks form a vital part of the policy development process. Significantly, as a means of learning about public concerns, the Department of Health and Human Services hosts forums, public hearings, and summits that allow community members to share ideas, with the aim of assisting the department in identifying areas for improvement [17, 18]. In the UK there is a declared government policy of establishing citizen panels and local advisory forums, and primary care trusts seek to connect with public opinion systems [19, 20]. Nevertheless, even in those countries in which the public is explicitly included in health-related policy, the government does not publicize the actual extent of public involvement or its impact on the process, nor is information available on contributors’ identity or motives [21].

To begin to address the lack of knowledge on public opinions regarding criteria for organ allocation, we carried out a survey to systematically measure such opinions among members of the Jewish-Israeli public. Specifically, we focused on allocation of kidneys, the organ type with the highest proportion of patients on waiting lists [22]. In Israel, as in most countries, deceased-donor kidneys are assigned to recipients on the basis of a patient-oriented point system. In line with the discussion above, this system takes into account medical efficiency considerations, coupled with various equity considerations designed to make transplantation accessible to as many patients as possible [23]. Medical efficiency is captured in factors such as blood group match, donor-recipient age difference, panel reactive antibody (PRA) level, and, importantly, immunological compatibility as reflected in human leukocyte antigen (HLA) matching: the number of “matches” and “mismatches” between the donor and the recipient in a set of six antigens. Equity considerations include waiting time (equity considerations in other, larger countries include the geographic location of the patient relative to the donor, and the national net kidney balance, i.e., calculation of import/export balance). We note that Israel is distinct from other countries in that, as a means of incentivizing individuals to register to donate, it has legislated a law that assigns priority status to candidates on the basis of their own or their first-degree relatives’ expressed willingness to donate organs [24, 25] (see Table 2 for details regarding this incentive system). It is unclear whether Israel’s official allocation criteria reflect community values.

**Table 2:** Priority points for willingness to donate

Extra points	Target population
3.5 points	*Organ donors* Waitlisted patients or a first-degree relative who actually gave their consent to procurement of organs from a deceased next-of-kin, or who donated an organ during their lifetime.
2 points	*Registered donors* Waitlisted patients who signed an organ donor card at least three years before they were added to the waiting list.
1 point	*Priority by affiliation* Waitlisted patients who are not registered as donors yet who have a first-degree relative who did sign an organ donor card at least three years before the patient was added to the list.

In our survey, we sought to achieve the following goals: (1) to evaluate the general public’s knowledge and understanding regarding organ transplantation in general, and kidney transplantation in particular; (2) to examine which criteria the community prioritizes in the organ allocation process—both in terms of declared preferences, and in terms of hypothetical allocation decisions (i.e., the criteria that individuals actually rely on when required to make a decision); (3) to assess whether taking public opinion into account in a kidney allocation policy has the potential to increase willingness to donate organs. In evaluating the results of the survey, we devoted particular attention to the question of whether current organ allocation policies are consistent with societal values and public preferences. We note that an abundance of surveys conducted in countries such as the US, the UK, and Australia have suggested that the vast majority of the public is willing to sacrifice some degree of medical efficiency of transplantation programs in exchange for an increase in equity or fairness in the allocation of donor organs [16, 26–28]. This paper seeks to discover whether Israel’s public holds similar perspectives.

We note that several studies have previously been conducted in Israel to shed light on various aspects of the public’s perception of organ donation. These studies investigated the attitudes of a specific group towards organ donation (college students [29]; the Zionist ultra-orthodox community [30]), examined public thoughts about directed organ donation to registered donors [31], studied the impact of particular personality variables on willingness to become an organ donor [32], explored factors that encourage or inhibit organ donation [7] or surveyed willingness to donate in exchange for prioritization in organ allocation [33]. In contrast to these studies, the current study focuses on the preferences and values of Israel’s Jewish population with regard to different aspects of the current organ allocation policy. Notably, this survey puts the public in the ‘driver’s seat’ and asks them to confront tough ethical questions. Furthermore, in contrast to previous surveys carried out in other countries, which focused on specific communities (e.g., scholars [27]; local community groups [28]; Arabic-speaking [34]), this survey seeks to represent Israel’s entire (Jewish) population, encompassing adult participants of all ages and from all walks of life.

This study’s premise is that an organ allocation policy is most likely to achieve an ethical balance between efficiency and equity when expert policy-makers allow public input to inform their decisions. Therefore, our objective is to provide insights that might contribute toward the development of such policies, which might consequently increase the community’s willingness to donate organs.

## Methods

We collected data through a telephone survey. A market research company—the B.I. and Lucille Cohen Institute for Public Opinion Research, an academic survey institute at Tel-Aviv University—administered a questionnaire to a representative sample of the general adult Jewish population in Israel. This particular survey did not include Arab citizens of Israel—see the “Limitations” section for further clarification. The inclusion criterion was being 18 years old or above. Respondents were sampled by a probabilistic sampling of households from strata of statistical areas, defined by socio-demographic characteristics of each area. Strata were designed to create homogeneity on the basis of geographic area (e.g., between large cities and small towns), immigration status (native-born individuals and established immigrants), level of religiosity (secular and orthodox) and socio-economic level. Sampling was done so that the probability of each statistical area to be included in the sample was proportional to the size of the population in the area. Such sampling ensures representation of various population groups, particularly those with a relatively small proportion.

The market research company approached 1438 households. Among these selected households, 300 people were unavailable when the survey was executed (i.e., they did not answer the phone call); 526 people refused to take part in the survey; and 8 participants only partially answered the survey questions, so they were excluded from the research. Therefore, the survey pool included 604 Israeli participants (42% response rate), ranging in age from 18 to 95 years (*M*_*age*_ = 49.31, SD = 16.76); half the participants were male.

The questionnaire, which appears in the Supplementary Material (Appendix), comprised 39 questions that were brief and straightforward. The questions addressed the following four categories: (i) familiarity with renal failure and treatment; (ii) declared preferences regarding criteria for organ allocation, and donation intentions regarding relatives; (iii) hypothetical decisions regarding criteria for organ allocation (participants were presented with “trade-off scenarios” in which they were asked to choose to allocate an organ to one of two hypothetical candidates with different characteristics; see Appendix); and (iv) demographic information and personal donation intentions.

We subsequently evaluated participants’ responses. Specifically, we produced descriptive statistics of participants’ characteristics and their priorities with regard to the allocation of donor organs, and we analyzed possible discrepancies between declared preferences and hypothetical allocation decisions. We used a Chi-square test to analyze nominal (categorical) data.

## Survey results

### Participant characteristics and familiarity with renal transplantation

Table 3 summarizes the characteristics of the survey population. According to participants’ responses, 75% of the respondents were born in Israel. Moreover, 55% of the participants stated that they define themselves as secular, 27% as traditional, 9% as religious, and 8% as orthodox. In our survey, 38% of the respondents stated that they hold a bachelor’s degree or higher, whereas 41% declared they have a high school diploma or elementary education. Thirty-seven percent of the participants (*n* = 226) declared they are organ donor-card holders. Other features that characterize the survey pool are as follows: 79% of the participants are parents to children, 34% have an above-average income, and 29% have below-average income.

**Table 3:** Characteristics of the survey population

Character		Percentage	Mean	SD
Gender	Male	50%		
	Female	50%		
Age	18–95		49.31	16.76
Country of origin	Israel	75%		
	Other	25%		
Religion	Secular	55%		
	Traditional	27%		
	Religious	9%		
	Orthodox	8%		
	Other	1%		
Parenthood	Yes	79%		
	No	21%		
Signed an organ donor card	Yes	37%		
	No	61.1%		
	Did not answer	1.3%		

### Awareness

Eighty percent of the participants specified that they were aware of most or all of the information presented at the beginning of the survey regarding the repercussions of renal failure and comprehended the customary treatment methods. Fifteen percent noted that they were aware of some parts of the information, while only 5% stated that they were not at all aware of the information.

### Declared preferences regarding criteria for organ allocation

**Fig. 3:**
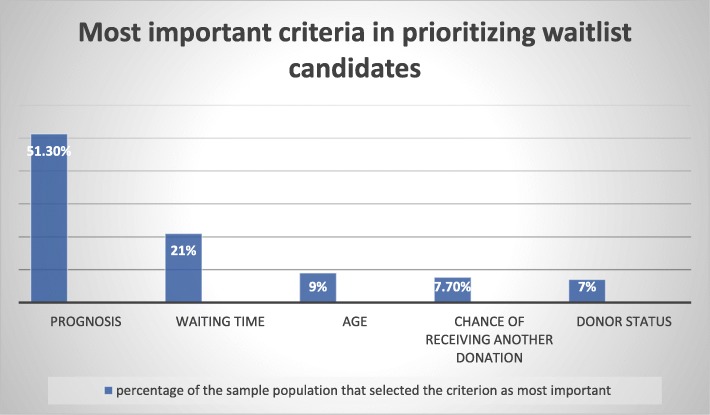
Most important criteria in prioritizing waitlist candidates, according to the survey population

Figure 3 shows which criteria were identified by the survey sample as most important in prioritizing waitlist candidates. The majority of participants (51.3%) selected prognosis/maximum benefit as the most important criterion, and the second most popular choice was waiting time (21%).

**Fig. 4:**
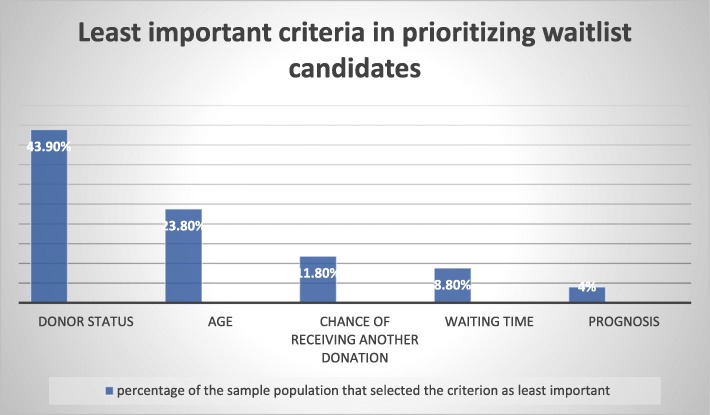
Least important criteria in prioritizing waitlist candidates, according to the survey population

When asked to identify the criterion that should be attributed the least significance in allocation decisions, 43.9% of respondents chose the recipient’s donor status (i.e., whether he/she is a registered donor). Contrary to expectations, respondents did not indicate that they perceived recipient age as a criterion that should be attributed high importance in organ allocation. In fact, 24% of the interviewees ranked it as the least important factor in determining transplantation priorities. Fig. 4 illustrates the criteria that were ranked by the survey population as least important.

Notably, the declared preferences of respondents who were registered donors were similar to the preferences of respondents who had not signed a donor card. Specifically, among registered donors, 43.2% chose the recipient’s donor status as the least important attribute to be considered. This finding is surprising in light of the fact that, as noted above, Israel’s allocation policy prioritizes registered donors, as an incentive to increase willingness to donate.

### Comparison between declared preferences and hypothetical allocation decisions

In most cases, participants’ declared preferences regarding organ allocation criteria were compatible with their hypothetical allocation decisions, as reflected in their decisions in the trade-off scenarios (presented in Table 4). In particular, analysis of the trade-off scenarios indicated that participants indeed assigned the greatest importance to prognosis (medical benefit) and waiting time. Moreover, their decisions confirmed that they did not assign substantial importance to age: For example, when asked to choose between giving a kidney to a 30-year-old person versus a 45-year-old person (with a similar prognosis; scenario 1 in Table 4), most respondents chose the option of “no preference” (71.5%). Notably, however, participants who did express a preference tended to prefer the younger candidate (*χ*^2^ = 45.62, *p* < 0.001). Moreover, participants significantly prioritized an older patient who had signed an organ donor commitment (36%) over a younger candidate who had not committed to organ donations (20%, *χ*^2^ = 27.75, *p* < .001, scenario 3 in Table 4). However, the age difference between the older and the younger candidates in our scenario was only 15 years. It is possible that including a larger age difference would have led to a greater preference for the younger candidate, indicating a discrepancy between declared preferences and hypothetical allocation decisions regarding the importance of age in kidney allocation decisions (see the “Limitations” section for a broader discussion of this idea).

**Table 4:** Comparison between the Israeli National Transplant Center’s point system and public preferences

Scenario #	Description	Survey results	The point system decision^a^	
1	Patient A is 30 years old. Patient B is 45 years old.	Patient A- 20% Patient B- 5% **No preference- 71.5%**	**Patient A** 2.9 points	Patient B 1.4 points
2	Patient A’s prognosis for a successful transplant is 70%. He has spent 4 years on the waiting list. Patient B’s prognosis for a successful transplant is 90%. He has spent 1 year on the waiting list.	Patient A- 39% **Patient B- 45%** No preference- 12%	Patient A 3.92 points	**Patient B** 4.48 points
3	Patient A is a registered organ donor. He is 40 years old. Patient B has not signed an organ donor card. He is 20 years old.	Patient A- 36% Patient B- 20% **No preference- 41%**	Patient A 3.9 points	Patient B 3.9 points
4	Patient A has a 70% chance of finding another suitable kidney in the coming year. He is a registered organ donor. Patient B has a 30% chance of finding another suitable kidney in the coming year. He has not signed an organ donor card.	**Patient A- 40%** Patient B- 36% No preference- 21%	Patient A 4 points	Patient B 4 points
5	Patient A is a registered organ donor. He has spent 1 year on the waiting list. Patient B has not signed an organ donor card. He has spent 4 years on the waiting list.	Patient A- 23% **Patient B- 53%** No preference- 21%	**Patient A** 2.48 points	Patient B 1.92 points
6	Patient A’s prognosis for a successful transplant is 70%. He is a registered organ donor. Patient B’s prognosis for a successful transplant is 90%. He has not signed an organ donor card.	Patient A- 27% **Patient B- 51%** No preference- 19%	Patient A 4 points	Patient B 4 points

^a^ In the case of a dilemma, in which the point system assigns equal scores to two or more patients (i.e., points are assigned for age, donor status, HLA match, PRA level), the National Transplant Center seeks expert advice in order to hold a medical debate and to decide which patient should receive the organ

Nevertheless, we did observe several discrepancies between declared preferences and the hypothetical allocation decisions. For example, when assigning scores to the various criteria, participants generally ranked maximum benefit (*M* = 6.52, *SD* = 1.09) significantly higher than they ranked waiting time (*M* = 6.21, *SD =* 1.32), (*t* (603) = 4.72, *p* < .002). Yet, when asked to allocate a kidney to one of two patients—patient A, who has a 70% chance of a successful transplant and has been waiting 4 years, and patient B, who has a 90% chance of a successful transplant and has been waiting only 1 year (scenario 2 in Table 4)—participants showed no significant preference for patient B (45% chose patient B; 39% chose patient A, *χ*^2^ = 2.56, *p* = .11). This decision seems to violate their own declared preference.

Moreover, as noted above, participants seemed to assign low priority to the candidate’s status as a registered donor, but their decisions in the trade-off scenarios revealed a more nuanced picture. For example, when rating the various criteria on Likert scales, participants assigned a significantly lower rating to donor status than to the likelihood of finding another kidney in the coming year (*M* = 5.0 vs. *M* = 5.9, *t* (603) = 8.4, *p* < 0.001). Yet, when asked to choose between a patient who was a registered donor with a 70% chance of receiving another kidney in the coming year versus a non-registered donor with only a 30% chance (scenario 4 in Table 4), participants showed no significant preference for one of the patients (40% of participants chose the former, while 35.6% chose the latter; *χ*^2^ = 1.06, *p* = .30).

### Willingness to donate the organs of a deceased relative

Participants’ willingness to donate a deceased relative’s organs was relatively high, 64% indicated that they would definitely agree, and 26% said that they would probably agree (but note that 16% did not want to answer this question) (*M* = 3.5, SD = 0.8; on a 4-point scale), with registered donors expressing significantly greater willingness to donate (*M* = 3.85; SD = 0.40) compared with participants who had not signed a donation commitment (*M* = 3.2; SD = 0.90; *t* (498) = 9.10, *p* < .001).

Interestingly, participants indicated that they would be significantly more willing to donate a relative’s organs if the criteria they had specified as most important were integrated into the allocation policy (*F* (1,489) = 9.53, *p* = 0.002). For example, among 48 respondents who were reluctant to donate their loved one’s organs (i.e., rated their agreement as 1- “I would definitely not agree to donate” or 2- “I would probably not agree to donate”), 18% expressed higher willingness to donate if their preferences were incorporated into the organ allocation policy.

### Willingness to sign an organ donation commitment card

As noted above, 61.1% of the participants were not registered organ donors. When asked about their willingness to sign an organ donation commitment in the near future, participants expressed relatively low intention to sign a commitment (*M* = 1.44; SD = .50, on a 4-point scale). However, when asked to rate their willingness to sign an organ donor commitment given that their preferences will be taken into account in the allocation policy, participants’ willingness to sign the card was significantly higher (*M* = 2.25; SD = 2.17, *t* (146) = 4.80, *p* < .001).

### Differences and similarities between the Israeli National Transplant Center’s point system and the Israeli Public’s preferences

Having characterized the Israeli public’s preferences regarding organ allocation criteria, it is interesting to compare these preferences to the actual criteria used by the Israeli National Transplant Center to allocate organs. Table 4 shows, for each of the six trade-off scenarios described in the “hypothetical decision” section of the questionnaire, the preferences expressed by survey respondents versus the actual decision that would have been made, based on the points granted by the Israeli National Transplant Center’s point system.

This comparison suggests that, in most cases, the views of the public are in line with those of the National Transplant Center. In particular, both the public and decision-makers assign high importance to maximum (medical) benefit and acknowledge the importance of waiting time. Nevertheless, we do observe some divergence. For example, the public attributes lower importance to recipient age than the point system does (scenario 1). The public’s decisions also illustrate their somewhat “fickle” perceptions of the significance of organ donation commitment: For example, in scenario 4, which traded off organ donation commitment against the likelihood of receiving another kidney, the public assigned *greater* weight to organ donation commitment than the point system did (and, as noted above, this decision seems to violate the public’s declared preferences). In scenario 6, which traded off organ donation commitment against waiting time, the public assigned *lower* weight to organ donation commitment than the point system did.

## Discussion

Participation in the survey was voluntary. Interviewees were not incentivized to take part in the research and were contacted during their free time. They were asked to confront hard questions, judge difficult scenarios, and share deep insights. We suggest that the fact that 604 people (42% response rate) from all walks of life decided to dedicate their time to tackle questions that are not mundane for the layman shows that the general public is interested in and ready to answer profound and complex questions regarding organ donation issues. This proposition is in line with findings of surveys of other populations (e.g., [27, 28]). Moreover, our findings suggest that people are interested in taking part in shaping organ allocation policies, as reflected in the fact that participants indicated that they would be more willing to sign an organ donation commitment and to donate the organs of a deceased relative if their opinions were taken into account. Encouragingly, we observed substantial common ground between the preferences of the public and the actual considerations used in Israel’s organ allocation system, suggesting that such public participation is feasible. We encourage the Israeli National Transplant Center to advise the public that the point system currently used already takes public preferences into account.

**Fig. 5:**
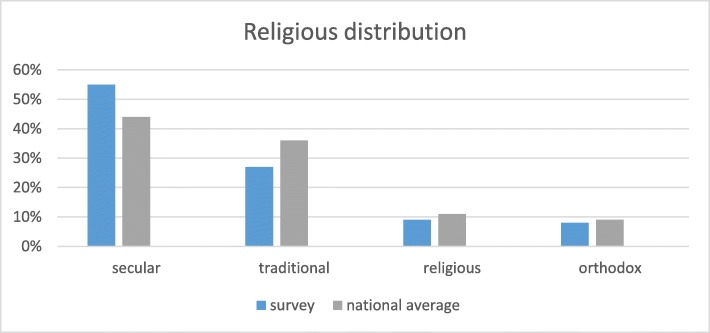
Distribution of religious affiliation in the survey population compared to the national average

In interpreting the survey’s results, it is important to acknowledge potential differences between Israel’s population distribution and the characteristics of the survey population. The extent of divergence varies across different parameters. Specifically, participants’ countries of origin are in line with those of Israel’s general population, with 75% of survey participants stating they were born in Israel [35]. Likewise, the distribution of survey respondents’ religious affiliations—which may have a substantial role in attitudes toward organ donation—also seems to reflect the actual distribution in Israel [35], which is as follows; 44% secular, 36% “traditional” (religious or not religious), 11% religious, and 9% orthodox (see Fig. 5).

**Fig. 6:**
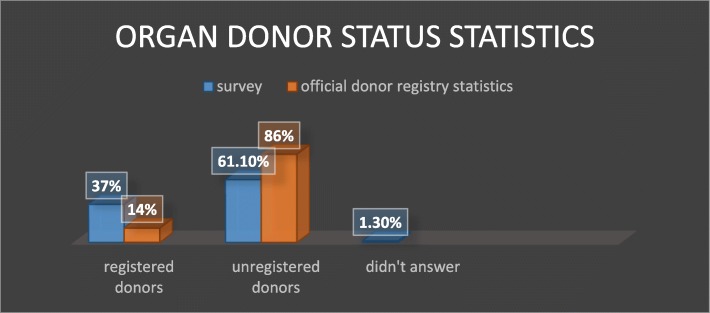
Comparison of donor status in the official donor registry versus in the survey

Respondents’ education levels and their likelihood of being registered donors diverge somewhat from the characteristics of Israel’s general population. According to the records of Israel’s Ministry of Finance [36], approximately half the population between the ages of 25 and 64 are academically educated (i.e., bachelor’s degree or higher), yet in the survey only 38% of the respondents stated that they hold a bachelor’s degree or higher. The deviation might stem from the fact that our survey pool includes respondents outside the age range of 25–64, including young adults who have yet to complete higher education, and elderly people who have accumulated life experience rather than academic education. The likelihood of being registered as a donor, in turn, was substantially greater among survey participants than among members of the general Israeli population (as illustrated in Fig. 6): 37% of survey participants declared that they are organ donor card-holders (*n* = 226), whereas only 14% of adults in the general Israeli population (944,849 individuals) have signed an organ donor card [6]. The over-representation of registered donors in our sample might have stemmed from the size of the survey (i.e., the number of people interviewed) or a sampling error.

Most of the characteristics of the survey population (i.e., gender, country of origin, religion) are in line with Israel’s population distribution and constitute a reliable representation of Israel’s Jewish population. Hence, the research findings and conclusions can be generalized and extended from the sample population to the population at large. Nonetheless, owing to the lesser levels of education of the survey pool and the over-representation of registered donors, the results might be a bit skewed. With that said, we suggest that these discrepancies do not detract from the results or lessen the importance of the conclusions stemming from this study (for a broader discussion of the limitations of our sampling procedure see also the “Limitations” section).

Like residents of other countries who responded to similar surveys [15, 37, 38], the Israeli public ranked maximum benefit/prognosis and waiting time as the most important criteria in the allocation process. The fact that respondents prioritized these criteria, which also direct the allocation policy of the National Transplant Center in Israel, proves that the public comprehends that allocation decisions hinge on a complex balance of medical efficiency and equity principles.

One interesting finding of the present survey, which diverges from findings of previous research, relates to the age criterion. In surveys conducted in the UK, Australia, and the US [27, 28, 37, 39], most respondents thought that in the name of fair innings or better prognosis, precedence should be given to younger patients. Apparently, the Israeli public does not hold the same opinion, since according to the declared preferences questions, 24% of respondents ranked age as the least important factor in organ allocation. Our observation might stem from several reasons such as cultural differences between Israel and the Western world, the life expectancy in Israel, which is 3 years higher than the OECD average [40], or Israel’s enduring struggles and loss of young lives due to wars, terror attacks, and military operations. These explanations could shed light on the respondents’ rationale and might elucidate why Israelis may not perceive age as a criterion that ought to be given precedence in organ allocation decisions. Nevertheless, future research is needed to examine the study’s speculations.

An additional notable finding is that, though Israel’s organ allocation policies prioritize registered donors, presumably as a means of encouraging donation, respondents (including registered donors) ranked donor status as the least important criterion in allocation decisions. This result diverges from outcomes obtained in surveys in Israel [7, 31, 33] as well as in other countries, in which respondents indicated that priority ought to be given to registered donors (in spite of the fact that their countries of origin do not prioritize on this ground [15, 39]). Nevertheless, it is important to note that when confronted with hypothetical trade-off scenarios, respondents did choose, in some cases, to prioritize a registered donor over a candidate who had not registered as a donor.

Indeed, as discussed above, we found multiple discrepancies between respondents’ declared preferences regarding organ allocation criteria and their hypothetical allocation decisions, as reflected in their decisions in the trade-off scenarios. In particular, our observations may point to a trend in which, when confronted with a specific case, participants consider medical efficiency to a lesser extent than they would theoretically prefer (as indicated in their declared preferences) and instead rely more heavily on equity principles such as waiting time or donor status. This tendency is in line with previous research suggesting that systematic discrepancies exist between ethical decisions that are made from an abstract, global perspective, and decisions that are made with regard to specific cases (e.g., [41–43]). Specifically, in decisions made regarding distinctive cases, decision-makers tend to show more flexibility and are inclined to give more weight to individuals’ particular characteristics and needs.

At first glance, our survey results might seem to suggest that people were more willing to donate the organs of a deceased relative than to sign an organ donation commitment card. However, a closer look reveals that in comparison to participants who had not signed a donation commitment, registered donors expressed considerably greater willingness to donate the organs of a deceased relative. Hence, the differences between the results are not substantial.

## Limitations

In spite of the study’s contribution to the understanding of the Israeli public’s preferences and beliefs regarding organ allocation, it has several limitations. First, the number of scenarios included in our survey was chosen according to the recommended length for a telephone survey. Obviously, these examples are limited and can only provide initial ideas regarding the directions of potential discrepancies between declared policy and specific allocation decisions. Second, we note that in the trade-off scenarios, participants were not forced to make a binary choice between the two candidates described; that is, they could select a “no preference” option and thereby avoid making a decision. Yet, in real life, due to organ scarcity, medical professionals do not have the privilege to avoid deciding between two equally deserving transplantation candidates. It would be interesting for future surveys to omit the “no preference” option and to observe participants’ responses when forced to make a choice, as in the real world. Third, the study’s population was composed of the general adult Jewish population in Israel. We decided to focus on the Jewish population since we assumed that cultural and religious differences might impact preferences and standpoints. The Arab citizens of Israel constitute about 20% of the population and are an integral part of the country, and therefore their stances and attitudes regarding organ allocation are of immense importance. Future studies ought to examine and evaluate Arab citizens’ values and beliefs. Fourth, since Israel is one of the first countries to implement non-medical criteria in organ allocation, the survey particularly focused on the population’s stance regarding the candidate’s status as a registered donor. In future studies, more balanced trade-off scenarios should be displayed. Fifth, when composing the trade-off scenarios, we did not notice that we had designed several scenarios in which, according to the point system decision, the score for both patients was identical (as illustrated in Table 4). We recommend that future studies pay attention to this detail in order to generate conclusive scores. Sixth, the survey was designed using probability proportional to size weighting for communities sampled. Nevertheless, owing to response bias, the demographics of the sample slightly deviate from the parameters of the general (adult, non-Arab, lay-Jewish population). This limitation of unweighted estimates might hamper the ability to draw far-reaching conclusions about public opinion. Nonetheless, it does not invalidate the study’s conclusions. Finally, transplant recipients can range in age from young children to elderly people. However, the trade-off scenarios described to participants referred to hypothetical patients within a limited age range (20–45). Future studies that rely on such scenarios should expand the age range in order to address policy or clinical questions that arise when elderly people are concerned.

## Conclusions

The results of this survey suggest that Israel’s citizens can articulate their preferences regarding organ allocation decisions. Respondents did not always consistently prioritize certain criteria over others; rather, their perceptions regarding which allocation criteria are most important depended, in certain cases, on the specific candidates under consideration. With that said, their preferences are generally compatible with current organ allocation policy. Moreover, participants indicated that they would be more willing to donate organs if allocation policies took their preferences into account. In general, people tend to be more inclined to give back to society (i.e., more willing to donate) when they feel that their own voices are heard. Accordingly, we propose that the public should be allowed to seek clarification, engage in discussions, express their views, and listen to other opinions and that in turn, decision-making entities should take community preferences into account when formulating their guidelines. Though we are aware that it is infeasible for a complex system to perfectly reflect the priorities of all stakeholders, ultimately, we propose that an optimal allocation system must strive to find a balance that respects community values and perceptions while maintaining continued clinical effectiveness [15, 44]. More broadly, we propose that any group of key stakeholders—including healthcare clinicians, patients, their families and carers, donor representatives, and the general public—is most likely to perceive an allocation system as efficient and equitable if the system takes into account that group’s views regarding which criteria yield efficiency and equity.

## Data Availability

All data generated or analyzed during this study are included in this published article [and its supplementary information files].

## Appendix

### Questionnaire

The questionnaire consisted of 39 questions that were brief and straightforward. The questions were divided into the following four sections.

*Section 1: Familiarity with renal failure and treatment.* In this section, participants were presented with a general explanation about renal failure and were then asked to rate their level of knowledge, understanding, and awareness regarding customary treatment options on a four-point scale (i.e., unaware, partially aware, mostly aware, and completely aware).

*Section 2.a.: Declared preferences regarding criteria for organ allocation.* In the second section, participants were presented with a set of patient-specific characteristics and were requested to rank, via a 7-point Likert-type scale (1 = not at all; 7 = very much), the extent to which each characteristic should be taken into consideration as a criterion in organ allocation decisions in cases of organ scarcity. The selected criteria were derived from a literature review of factors that have been shown to influence organ allocation decisions and included both efficiency and equity principles. Specifically, participants rated the importance of the following characteristics: the candidate’s age, the amount of time he or she has spent on the waiting list, having dependents/family responsibilities, donor status (i.e., whether or not the candidate is a registered donor), likelihood of receiving another donation in the coming year, and prognosis/maximum benefit. Next, they were asked to select the most important criterion, the second most important criterion, and the least important criterion. In addition, participants were asked to specify any additional criteria which, in their view, should be taken into account in decisions about how donor kidneys should be allocated.

*Section 2.b. Donation intentions (relatives).* After expressing their preferences regarding organ allocation criteria, participants were asked to rate (i) the extent to which they would be willing to donate the kidney of a deceased relative, and (ii) the extent to which they would be willing to donate the deceased relative’s kidney if their preferences (as expressed in section 2.a.) were incorporated into the allocation policy (each on a 4-point scale).

*Section 3: Hypothetical decisions regarding criteria for organ allocation.* In this section, participants were presented with six hypothetical “trade-off” scenarios, in which they were requested to place themselves in the role of the decision-maker. In each scenario, two different candidates with different characteristics were described. The respondent had to select which of the two should receive a kidney or select “no preference.” This section enabled us to determine which criteria respondents actually prioritized when forced to make a decision (the six scenarios are described in detail in the section titled “Differences and Similarities …” ). In particular, to examine public opinion regarding the law according to which a person can gain priority points by registering as an organ donor, three scenarios evaluated whether respondents prioritize organ donation commitment over other criteria, namely, age, waiting time, and chances for a successful transplant.

*Section 4: Demographic information and personal organ donation intentions*. Respondents provided demographic information, including their age, education, religion, country of origin, marital status, socioeconomic status, and number of children. In addition, they were asked whether they were holders of an organ donor card and if they or their relatives had ever received an organ transplant. Furthermore, respondents were asked to rate the extent to which they were willing to sign an organ donor card in the near future, and the extent to which they would be willing to do so if their preferences were incorporated in the allocation policy, both on a four-point scale (definitely not, probably not, probably yes, definitely yes).

## Abbreviations

HLA: Human leukocyte antigen
OPTN: Organ Procurement and Transplantation Network
PRA: Panel reactive antibody
